# Personal experiences following acquiring HIV infection while volunteering in Phase I/II HIV vaccine trials: A qualitative study from Tanzania

**DOI:** 10.1371/journal.pone.0276404

**Published:** 2022-10-26

**Authors:** Edith A. M. Tarimo, Joel Ambikile, Patricia Munseri, Muhammad Bakari

**Affiliations:** 1 Department of Nursing Management, Muhimbili University of Health and Allied Sciences, Dar es Salaam, Tanzania; 2 Department of Clinical Nursing, Muhimbili University of Health and Allied Sciences, Dar es Salaam, Tanzania; 3 Department of Internal Medicine, Muhimbili University of Health and Allied Sciences, Dar es Salaam, Tanzania; Torrens University Australia, AUSTRALIA

## Abstract

**Background:**

Participation in HIV vaccine trials is an essential step towards development of an effective preventive vaccine. A Phase I/II HIV vaccine trial enrolls volunteers at low risk of acquiring HIV infection, however a few may still become infected. Understanding the experiences of volunteers who acquired HIV infection while participating in such trials is essential for future research. Here, we describe experiences of HIV infected volunteers in Phase I/II HIV vaccine trials conducted in urban Tanzania.

**Materials and methods:**

We used a case study design. In-depth interviews were conducted with four participants who became HIV infected during long follow-up visits after completion of vaccination schedules in a Phase I/II trial. Between 3 and 8 years after HIV positive diagnosis, each participant was interviewed at three time points within a two-year interval so as to allow for accumulation of experiences and cross-checking the emerging constructs. Data was analyzed using a qualitative data analysis framework.

**Results:**

Analysis revealed that participation in HIV vaccine trials involves balancing controversies and the spirit of informed decision. The participants declared that they did not acquire HIV from the experimental vaccine. Disclosure of HIV status within the family was gender specific. Men were hesitant to disclose their HIV status to their sexual partners fearing for the consequences. Women’s attempt to disclose their HIV status yielded negative reactions from the sexual partners. The acquired knowledge from the HIV vaccine research enabled the participants to cope with the uncertainties and their health status.

**Conclusions:**

The knowledge acquired during the Phase I/II HIV vaccine trial appears to be an essential resource to cope with uncertainties post research. The HIV vaccine trial implementers need to understand the challenges the volunteers may confront after the trial while coping with their health status. Longitudinal studies are essential to trace the effects of uncertainties to the individual participants.

## Introduction

Human Immunodeficiency Virus (HIV) continues to devastate the health system in some parts of the world. Approximately 37.7 million people were living with HIV infection in 2020. In addition, there were 1.5 million new infections and 770,000 AIDS-related deaths in the same year despite widespread rollout of antiretroviral therapy (ART) [1]. In Latin America, age and geographical areas with large population were found to significantly determine the HIV related mortality [2]. Similarly, in sub-Saharan Africa, a substantial variation in HIV mortality rates between and within countries has been reported [3]. The most promising scientific approach to control and eventually end the HIV epidemic is availability of an effective, safe, cost efficient, and easily accessible worldwide preventive AIDS vaccine [4]. The initiatives to conduct HIV vaccine trials started way back in 1980s. The first phase I HIV vaccine trial was conducted in the USA in 1987 [5], and to date several HIV candidate vaccines have been tested [6].

A growing body of literature underscores the efforts of conducting HIV vaccine trials in different parts of the world [7], but the conduct of such investigations involves multiple scientific hurdles. The results from VAX004 trial conducted in America and Thailand in 1998 and 2003 did not reveal protective effect of HIV-1 infection [8, 9]. In Thailand, a large community-based randomized, double-blind phase 3 efficacy trial famously known as RV144 showed limited efficacy (31.2%) [10, 11]. Based on RV144 trial results, a large phase IIb/III HIV vaccine clinical Trial (HVTN 702), conducted among young adults in South Africa was prematurely ended in February 2020 due to the lack of efficacy in the prevention of HIV-1 among the vaccine group [12].

The most common scientific factors hindering the progress of HIV vaccine development include lack of natural immunity to HIV, variability of HIV types, lack of correlates of protective immunity, and lack of an animal model that reliably predicts vaccine efficacy in humans [13–15], and perhaps complex interactions between the genes, immunity and environment where individuals are heavily exposed but not acquiring HIV infection [16]. Despite the challenges faced in the HIV vaccine development [17], more trials are being conducted for eventual development of an effective preventive vaccine. Of the 20 trials conducted in South Africa amongst adults, three-quarters (15/20) were conducted with participants who were at low risk of HIV acquisition [18]. Social behavioral studies have been recognized to complement the conduct of HIV vaccine trials. In South Africa avoiding HIV risk was the main motivation to participate in clinical trials, followed by a desire to help the community or helping to find a vaccine [19].

Tanzania is one of the low middle-income countries conducting HIV vaccine trials to contribute in vaccine development. The first phase I/II HIV vaccine trial, the HIV Vaccine Immunogenicity Study-03 (HIVIS-03) conducted in 2007, reported a broad immune response among volunteers [20]. Based on the results from HIVIS-03 trial, two additional Phase I/II HIV vaccine trials, the Tanzania and Mozambique Vaccine trials (TaMoVac I&II) were conducted between 2008 and 2015 to evaluate safety and immunogenicity [21]. Another HIV vaccine trial (HIVIS-06) increased knowledge about the outcomes of different ways of administering the experimental vaccines [22, 23]. However, the process of developing an effective preventive HIV vaccine also depends on the willingness and voluntary decision of potential volunteers.

The willingness to participate in HIVS-03 vaccine trial amongst low risk population of Police Officers was associated with unrealistic intentions such as having more sexual partners [24]. Those who enrolled and completed the Phase I/II HIV vaccine trial appreciated the gained opportunities such as reduced risky sexual behavior, overcoming fear of HIV testing, privilege of complete medical examination, and ability to change to better health-seeking behavior [25]. A follow up study in this cohort of volunteers revealed that participants were more likely to have a sexual partner other than the spouse or cohabitant [26]. The findings from the above follow-up study [26] predicted that volunteers at low risk may actually be at risk for acquiring HIV for an unpredicted period of time. Following this assessment, it was deemed important to follow-up a sub-set of the participants who took part in the Phase I/II HIV vaccine trials and got HIV infection. We hereby describe the experiences of four participants who acquired HIV infection while volunteering in the Phase I/II HIV vaccine trials in urban Tanzania.

## Materials and methods

### Ethics statement

The study was approved by Muhimbili University of Health and Allied Sciences Research Ethics Committee (Ref. No.2015-01-15/AEC/Vol.IX/44; Ref. No.2016-02-18/AEC/Vol.X/180; Ref. No.2017-04-28/AEC/Vol.XII/82; Ref. No.DA.282/298/01.C/). Written consent was obtained, and the potential participants were assured of their freedom to withdraw from the study at any time. Confidentiality was assured and privacy was observed. No names were recorded in the transcripts.

### Study design

The study adopted a case study design [27]. This design is relevant because it generates an in-depth and a multi-faceted understanding of a complex issue in its real-life context [28].

### Participants

The participants were volunteers who were enrolled in Phase I/II HIV vaccine trials [HIVIS-03 and TaMoVac-I] [21, 29]. The eligibility criteria for enrolment in the trials included an age range between 18 and 40 years old; willingness to stay in the study for the stated period of 24 months; avoidance of pregnancy and use an effective contraceptive method for the duration of the trial among women; agreeing to HIV testing and adhering to condom use to prevent HIV acquisition, sexually transmitted infections and to avoid pregnancy [25]. The HIVIS-03 and the TaMoVac-I trials enrolled 60 and 80 volunteers respectively. Whereas the volunteers in HIVIS-03 were recruited from the Police force, the TaMoVac trials recruited participants from the Police force, Prisons force and the general community.

**Fig 1:**
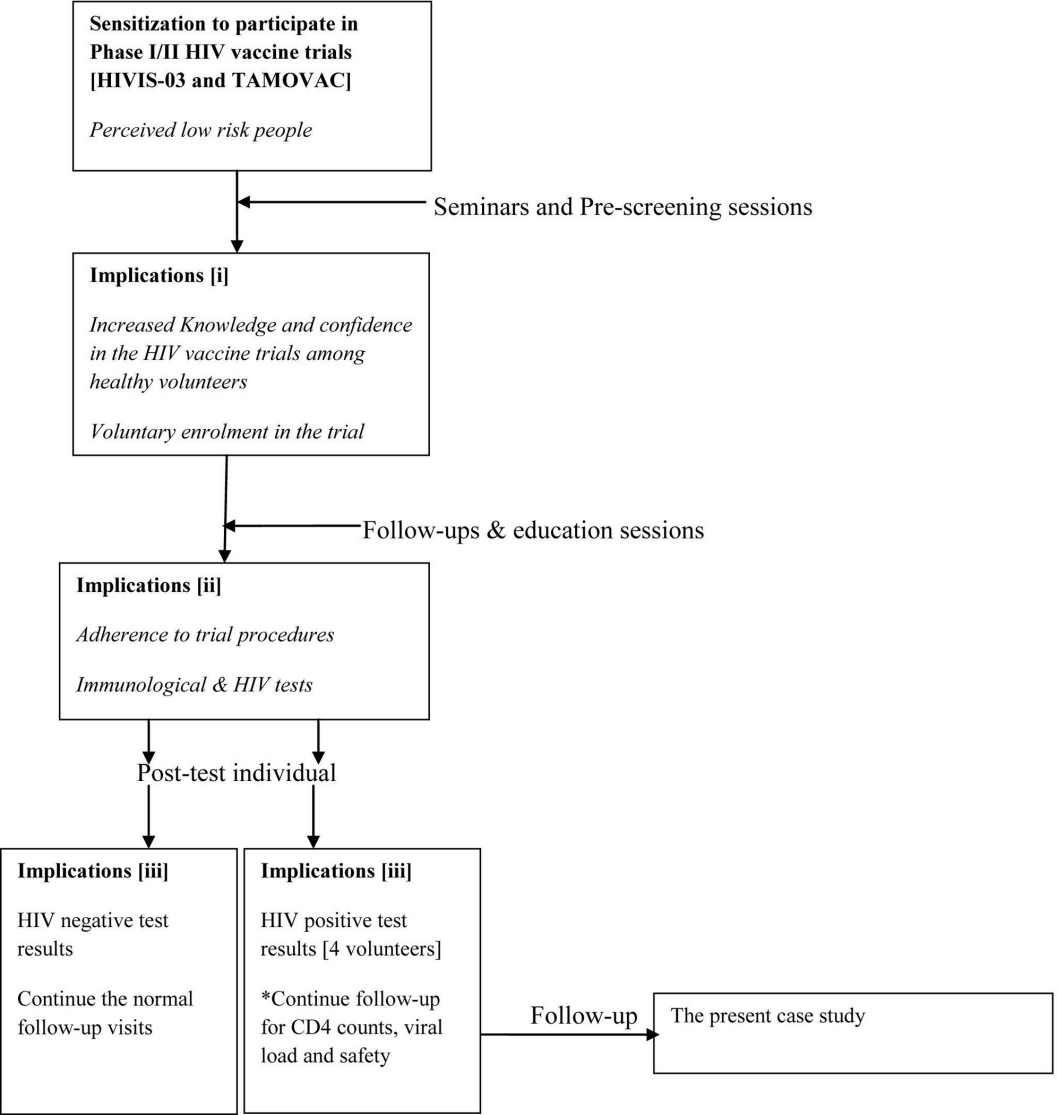
The participants’ journey in HIV vaccine trials.

During enrollment into a trial, the participants received detailed information about the trial conduct to enable them make informed voluntary decision to participate in the study. The principles of Good Clinical Practice were adhered to throughout the trial conduct. Furthermore, volunteers received intense education on measures to prevent HIV acquisition. Per study protocol, infected volunteers were withdrawn from further immunizations but were followed up for safety. The participants’ journey from sensitization meetings to enrolment in HIV vaccine trials included the follow-up for CD4 counts, viral load and safety (Fig 1). The HIV infected volunteers were counseled, and referred to appropriate HIV care and treatment centers.

Five volunteers acquired natural HIV infection that was confirmed through a Polymerase Chain Reaction (PCR). The HIV infection was acquired during long follow up visits after completion of vaccination schedules. The planned vaccinations and boost were completed during visit 12, and the participants were found to be HIV infected during visit 19. The long follow-up ended in the 24^th^ visit. The enrolled participants for this study were thus from two sources (HIVIS-03 and TaMoVac-I), and the interviews took place 3–8 years after the long follow-up visit.

### Data collection tools

Authors developed the key questions based on the knowledge gained from socio-behavioral components of the respective HIV vaccine trials to conduct in-depth interviews (IDI). These were used to develop a data collection tool. However, the tool did not directly focus on the experiences following acquiring HIV infection. Thus, a semi-structured interview guide contained the following key questions:

- What is your experience of participating in the HIV vaccine trial?
- What are your opinions about the rumors that volunteers in HIV vaccine trials were implanted with HIV?
- What are the researchers’ [HIVIS-03 and TaMoVac investigators] role towards the HIV infected volunteers after the study?

The guide was modified to accommodate emerging issues as follows:

- Please, tell me what you do from morning to evening
- Please, describe your decision to enroll in the HIV vaccine trial. What motivated you to participate?
- In your own words, what did you benefit from participating in the HIV vaccine trial?
- What challenges are you experiencing now due to participation in the HIV vaccine trial?
- Participation in the HIV vaccine research has been a long a journey. Please, in your own words, tell me any unusual events that you encountered in this journey.

### Procedures

#### Entry into the field

In the present study, the infected volunteers were contacted, informed about the purpose of the study, and were scheduled for an interview that was conducted by the first author (EAMT) who worked as a Social Scientist for the vaccine trials and was familiar with the trial procedures. EAMT, a female with PhD and a Lecturer at the Muhimbili University of Health and Allied Sciences (MUHAS), has been trained in qualitative research methods and has been teaching short courses on qualitative research methods to both undergraduate and postgraduate students for more than three years. She participated in the weekly research meetings and conducted a series of research in the HIVIS-03 project [24–26, 30–35]. Participants for the current research were purposefully recruited through the HIV vaccine trial clinic at MUHAS in Dar-es-Salaam, Tanzania. Of the five HIV infected volunteers, one was not recruited in the study because she was on studies outside Dar es Salaam during data collection. All interviews were conducted at the HIV vaccine trial site in the counselor’s room. Privacy and confidentiality were assured and the participants were comfortable.

#### Data collection, analysis and writing

We used the above IDIs guide to collect data face-to-face. Data were collected in three rounds at two years’ interval (June 2015, July 2017 and August 2019). The intervals of the interviews were estimated by the researchers to allow accumulation of experiences and changes that might have occurred regarding their health status. The interviews were conducted in Kiswahili, the national language. Twelve in-depth interviews that were audio-recorded were conducted with the participants after written informed consent was obtained. Informed consent was discussed in detail at the beginning of each interview with an emphasis on confidentiality, audio-recording, and the permission to disseminate the results anonymously. After signing the consent, interview and audio-recording commenced. Study numbers were used for each participant to protect their identity. After each interview, the audio-recorded information was transcribed verbatim. During the third round, the IDIs guide was modified to deepen the participants’ prolonged experiences notwithstanding their HIV positive status. Field notes were made after the interview. The duration of interviews ranged between 20 and 38 minutes. After each interview, a Research Assistant who was a medical student transcribed all the audio recorded data verbatim. Participants were given a summary of the previous conversation prior to the next interview as a means of getting feedback. Data saturation was noted during the second and third round interviews where most of the information appeared repetitive.

EAMT (BScN, MPhil., PhD) was the in charge of data analysis. EAMT and JSA (BScN, MSc.) read the raw textual data and independently coded all the transcripts. Analysis was carefully done manually to avoid losing meaning of the participants’ expression. The five steps of qualitative data analysis framework were adopted as follows: (i) familiarization with data through reading each transcript and writing labels to the data; (ii) identification of a thematic framework by jotting down key concepts that were re-emerging from the data; (iii) indexing the data by listing the open codes that had been made to the data; (iv) charting data through arranging appropriate thematic references in a chart which enabled comparison across transcripts and within each transcript; and (v) mapping and interpretation of the data through which data were examined and interpreted [36]. These five steps were loosely applied to represent the transparency of data analysis. We also applied interpretive description (ID) approach [37] to provide contextual understanding to guide future research on the targeted population. It is known that ID generates new insights on what may characterize the experiences of individuals who enroll in HIV vaccine trials and get infected in a similar context.

## Results

The study comprised of four participants with age ranging from 25 to 43 years. The participants enrolled were two women (one married with two children and the other separated with one child) and two men, both men were married with two and three children respectively. Participants’ education levels ranged from seven years of primary education to college education (Table 1).

**Table 1:** Socio-demographic characteristics.

SN	Sex	Age	Education	Marital status	No. of children	Name of trial, year of Enrollment	Participant identity in this research
1	F	43	College	Married	2	HIVIS03 2007	P1, Older woman
2	F	25	Standard seven	Separated	2	TAMOVAC-01, 2012	P2, Young woman
3	M	37	Standard seven	Married	3	HIVIS03 2007	P3, Older man
4	M	34	Form IV/College	Married	2	HIVIS03 2007	P4. Young man

Two themes emerged and seven categories were constructed from the data and these are summarized below (Table 2).

**Table 2:** Themes and categories.

SN	Themes	Categories
	Balancing controversies and the spirit of informed decision post HIV vaccine trial	Decision to participate in an HIV Vaccine Trial
		Altruism
		Disclosure of HIV status
	Coping with HIV post the HIV vaccine trial	Knowledge about the HIV vaccine research
		Gained benefits from research participation
		Self-acceptance
		Post-trial connections

### Balancing controversies and the spirit of informed decision post HIV vaccine trial

A strive to balance the decision to enroll in HIV vaccine trials and emerging negative comments from the surrounding communities was essential as described below:

#### Decision to participate in an HIV vaccine trial

It emerged that the decision to participate in the HIV vaccine trial was largely a balancing act of controversies rampant in the community and the spirit of informed decision The rumors and misperceptions about the HIV experimental vaccine that participants in the trials were implanted with HIV generated nuisances in the community. While struggling to protect their identity, some of the participants were demoralized because of the rumors. One participant narrated:

*Whoever I try to explain about this [HIV vaccine trial]*, *truly I end up with a broken heart …truly*, *if you listen to people and you have not started [enroll in HIV vaccine trial]*, *you will not start…(P2*, *Young woman*, *1*^*st*^ *interview)*

Self-confidence and knowledge about the HIV vaccine trials were helpful to deal with the rumors in the surrounding community. The following voice confirmed the knowledge about the HIV vaccine trials:

*There are rumors*, *‘those [volunteers] have been implanted with the virus’ and so forth; it is all over; … those are people who did not get this knowledge [HIV vaccine trial education]… it depends on how the targeted individual [volunteer] responds because he/she has been educated and understand what was done (P4*, *Young man*, *1*^*st*^ *interview)*

Although the rumors from the surrounding community did not change the participants’ confidence in the HIV vaccine trial, some participants experienced discomfort, but the knowledge acquired during the vaccine trial suppressed the effect of the rumors as narrated below:

*I understand I was not implanted with the virus*, *but when people talk about it [being implanted with HIV] I get upset … when they point fingers to me*, *I must get upset*, *but I ignore because they [community] don’t understand what happened (P2*, *Young woman*, *2*^*nd*^ *interview)*.

The rumors from friends and relatives concerning the perceived negative effects of the experimental vaccine, did not alter the participants’ self-confidence.

*I did not listen to people’s words*. *I did not listen because I had made a decision to participate*. *It was voluntary (P1*, *Older woman*, *1*^*st*^ *interview)*.

#### Altruism

The altruistic intention to do good for relatives, friends and next generation was embraced as a priority in the HIV vaccine clinical trials.

*I participated believing that we were doing research to save the next generation because I am not the last generation (P4*, *Young man*, *1*^*st*^ *interview)*.

The participants were of the opinion that contribution to research was not only the moral obligation but achievement of the scientific goals. Thus, awareness of the research benefits appeared to fuel the decision to participate in the HIV vaccine trials. They said:

*If everyone steps back*, *who will be responsible*? *So personally*, *I have sacrificed to make the future for the family*, *grandchildren and the country… (P3*, *Older man*, *3*^*rd*^ *interview)*

*The target is to succeed getting a vaccine to save either myself*, *relatives*, *family or the next generation to use the vaccine because I see people perishing due to this disease [AIDS] (P1*. *Older woman*, *3*^*rd*^ *interview)*

#### Disclosure of HIV status

The analysis revealed gender differences in disclosing HIV status, as a new insight in understanding how gender influences the sharing of sensitive information. For women, disclosure of HIV status cultivated disharmony in marital relationships. In addition, uncertainty about the source of HIV infection led to refusal of joint voluntary counseling and testing. One woman said:

*Whenever I ask my husband to accompany me for an HIV test*, *he refuses…He tells me to go and get tested alone*, *and my results will reflect his HIV status*. *Truly*, *he is the most disruptive person (P1*, *Older woman*, *2*^*nd*^ *interview)*.

On the other hand, independent HIV testing and results disclosure resulted into marital separation to the other woman.

*The 1*^*st*^ *day of my HIV positive results*, *I disclosed to him [spouse]… That was when the quarrel began; I was offended*. *I decided to separate (P2*, *Young woman*, *1*^*st*^ *interview)*.

For men, disclosing the HIV status to the sexual partner cultivated harmony. Tolerance, caring attitude and unconditional acceptance of spouse’s HIV status characterized the woman’s response when the man disclosed his HIV status.

*She [wife] is understandable*, *tolerant*, *good caretaker*. *If I want sex*, *I have to be tricky not to infect her (P3*, *Older man*, *3*^*rd*^ *interview)*.

Sometimes men appear to keep their HIV status confidential to ensure harmony in their marital relationship. Time to disclose the HIV status was unpredictable though sometimes life went on without precautions to protect the sexual partner.

*I think time is not yet to disclose my HIV status*. *I see its better not to disclose*. *I don’t see a problem*. *I feel normal and I am progressing well (P4*, *Young man*, *3*^*rd*^ *interview)*.

Disclosure of HIV status appeared to be personal than a shared matter among men. Keeping confidential all matters that appear to cause psychological disturbance if disclosed was preferred. Thus, disclosure of HIV status heavily depended on the individual decision and perception.

*Everybody decides how to live*. *How life suits him without causing harm*. *If you live uncomfortably*, *it may confuse and harm you… This is my journey [Living with HIV]*. *I know what to do for the time being (P4*, *Young man*, *3*^*rd*^ *interview)*.

For both men and women disclosure of parental HIV status was considered sensitive and needed proper timing to avoid psychological effect to the children.

*I am waiting for the child to mature so that I can tell her*. *Eleven years old is still young to understand [HIV status] (P2*, *Young woman*, *3*^*rd*^ *interview)*.

*Children are still schooling*. *Although they may understand*, *I don’t want to involve them in difficult issues [HIV status disclosure] before time (P1*, *Older woman*, *3*^*rd*^ *interview)*.

The participant who had three children; the first, second and third born studying at College, secondary school and grade six respectively shared similar concern:

*They [children] don’t know that I am ill*. *I don’t want to tell them*… *They are busy schooling…I don’t want to stress them…Although they may suspect [ill health] (P3*, *Older man*, *3*^*rd*^ *interview)*.

### Coping with HIV post the HIV vaccine trial

A number of concepts were critical in aiding coping with the HIV infection after the trial. These included:

#### Knowledge about HIV vaccine research

The knowledge acquired about the conduct of the HIV vaccine trials emerged as an opportunity to understand the research better. The seminars during the trial enabled the participants to defend their decision as research volunteers. Thus, knowledge was used to fight against negative perceptions regarding the experimental vaccine. They said:

*You know when you understand what is being done and hear people talking about it*, *it does not affect me because they don’t understand… It is different from me who knows what is being done (P4*, *Young man*, *2*^*nd*^ *interview)*

*I gained a lot of knowledge that will help me in my life …(P2*, *Young woman*, *1*^*st*^ *interview)*

Sometimes the intention to educate the community provoked mistrust in the sense that the trial team was not trusted. Nevertheless, this mistrust rarely affected those who participated:

*That rumor does not affect me*. *People construct the rumor that we are infected because of participating in the vaccine trial*. *Personally*, *I consider that person as uneducated about the current progress in the world… (P3*, *Older man*, *2*^*nd*^ *interview)*

The increased knowledge about the HIV vaccine trials assured the participants about the safety of the experimental vaccine. The safety of the HIV vaccine trial was evidenced verbally in the sense that the participants believed their HIV infection occurred due to risky sexual behaviors, and not the vaccine. Knowledge about the HIV vaccine trials shined out as the most beneficial aspect of participation in the HIV vaccine trials. Confidence of sharing HIV and vaccine facts confirmed the benefits obtained from participation in HIV vaccine trials. This knowledge facilitated dissemination of relevant facts about HIV vaccine trial to the community.

*I learned many things about HIV including the vaccines*. *That means I was able to educate those with misperceptions (P1*, *Older woman*, *2*^*nd*^ *interview)*.

On top of the acquired knowledge, the perceived preventive effect of the vaccine was embraced. Regardless of the source of HIV infection, the delay of the seriousness of the illness was regarded as the beneficial effect of participation in the trial.

*I have been living with it [HIV infection] for almost 10 years*. *This is 11*^*th*^ *year*. *The body started shaking [signs of illness] in 2019*. *I did not expect to be in this decade (P3*, *Older man*, *3*^*rd*^ *interview)*.

#### Gained benefits from research participation

Participation in HIV vaccine trials enabled the establishment of social networks and increased awareness about the individuals’ health status. Having health insurance facilitated regular health check-ups and eventual awareness of one’s health status complemented education on healthier life style. The perceived good communication with the trial team helped the participants in coping with their health status. Meeting the trial staff without hindrance assured the participants availability of professional support when they encounter health problems. One participant said:

*I did not know Dr Y [pseudo name]*, *even you [interviewer]*. *If I have a serious health problem*, *I have a possibility to take transport*, *and I know where to go*, *where to start*. *If I have a phone number*, *I can just call (P2*, *Young woman*, *3*^*rd*^ *interview)*

#### Self-acceptance

Overtime, normal life continued regardless of the participants’ HIV status. Time spent at work exceeded home hours. Feelings of happiness, free from worries and perceived self-satisfaction dominated the participants’ lives. The persistence of noises against the vaccine trial did not upset the participants.

*I have not given up… Time has passed*. *Even those noises [negative perceptions about the vaccine] have gone down (P3*, *Older man*, *3*^*rd*^ *interview)*.

The recurrence of illness was perceived as part of normal life despite the negative perceptions from the surrounding communities towards the HIV vaccine trial.

*Whatever I experience is normal*. *If I get fever*, *I get treatment in the hospital*. *It [fever] can happen to anybody regardless of participation in the research (P3*, *Older man*, *3*^*rd*^ *interview)*.

HIV infection was normalized in the context of believing in the protective effect of the experimental vaccine. The delay to start ARVs amongst all the participants reflected the perception of protective effect of the vaccine. In addition, the delayed initiation of ARVs was facilitated by the uncertainty of disclosing the HIV status to sexual partner.

*I wanted to start treatment [ARVs] earlier*. *It was difficult to involve her [wife]*. *For almost 4 years*, *she does not know my status (P3*, *Older man*, *2*^*nd*^ *interview)*.

Also, the low levels of viral load delayed the initiation of ARVs amongst the participants. The repeated viral load tests assured the participants of their wellness. The intention to start treatment was predicted by viral load levels and physical fitness.

*I wanted to re-test to see if I was supposed to start treatment; however*, *I have not experienced any health problem (P1*, *Older woman*, *2*^*nd*^ *interview)*.

#### Post-trial connections

The participants appreciated the investigators’ close follow up. They reported that regular contacts with the investigators facilitated health check-ups. Instead of starting new health check-up consultation, the continuation of health services at the trial site was considered helpful. Feedback about research progress, updates about the HIV vaccine development, and support to the infected participants through counseling were post trial essential participants’ wishes. They said:

*When people get infected while participating in the research*, *they should get counseling*… *Counseling is a small word*, *but it carries a lot of weight*. *You cannot even compare it [counseling] with money… (P1*, *Older woman 1*^*st*^ *interview)*

The meetings between trial implementers and post-trial participants were anticipated to increase knowledge and support:

*It is good to maintain regular communication; it will remind them [HIV infected] something…however; when you leave them for a long time; they will forget [the gained knowledge]; few people are aware of themselves that I am HIV infected*, *and thus ‘I am supposed to take care of myself to prolong my life…’ (P3*, *Older man*, *1*^*st*^ *interview)*

*… We were supposed to meet regularly*, *but we are not meeting; we have lost connections for a long time*. *Even to sit and discuss something is impossible…You know even this interview has brought a sense of comfort*, *when I talk to you 2–3 words*, *I gain comfort (P2*, *Young woman*, *2*^*nd*^ *interview)*

Moreover, the participants appreciated knowledge gained, adherence to research ethics, and customer care from the investigators during the trial.

## Discussion

In this study, two themes emerged. The first theme, ‘Balancing controversies and the spirit of informed decision post HIV vaccine trial’ details participants’ decision to participate in an HIV vaccine trial, the altruistic intention and how they dealt with disclosure of their HIV status. The second theme, ‘Coping with HIV post the HIV vaccine trial’ is supported by the participants’ knowledge about the HIV vaccine research, gained benefits from research, self-acceptance and the participants’ wishes post-trial. Overall, the participants encountered negative comments from the surrounding communities that they were implanted with HIV while participating in HIV vaccine trials, though this was a general concern irrespective of the HIV status. However, they used the knowledge acquired during the HIV vaccine trial participation to defend their decision against the community’s negative comments and that the experimental vaccine was safe. Also the participants faced difficulties to disclose their HIV status to the sexual partners because of fear of negative consequences.

The fact that participants encountered negative comments from the surrounding communities suggests lack of adequate information about experimental HIV vaccine safety among the community members surrounding the participants. Limited knowledge about HIV vaccine trials in the setting where these clinical trials were conducted in Tanzania has been reported. The earlier study in the same cohort revealed that people declined to enroll in HIVIS-03 trial because of fear of adverse outcome and negative comments from other people [30]. Also the study among the volunteers who enrolled and completed the HIVS-03 trial visits indicated that participants encountered discouragement from the colleagues and the rumors that the vaccine wasn’t safe [25]. A follow up study in the communities where the volunteers were recruited from confirmed the belief that participation in HIV vaccine trials was unsafe [34]. The fact that the above studies were conducted in series, without an intervention, the findings from the present study are not surprising. The demand of HIV vaccine knowledge has been reported from various places. In Kenya, a desire to receive education about HIV was a motivation for participation in HIV vaccine trials among MSM [38]. In high income countries, participation in HIV vaccine trials has been hindered by negative reactions from friends, family, and partners [39] probably due to lack of adequate information about the clinical trials. A systematic review among the minority ethnic groups suggests that future clinical trials should engage community stakeholders in all stages of the research process through community-based participatory research approaches [40].

In this study, the discomfort to disclose HIV status to the sexual partners implies that sharing health problems is a personal matter because of prevailing stigma and discrimination in low and middle-income countries. A systematic review indicates that HIV can impose stress, fear, anxiety and depression to infected individuals as well as stigma and discrimination [41]. In the USA, some study participants chose not to disclose their status to anybody because of fear of detrimental outcome towards their own well-being [42]. HIV disclosure appears a complex process in which an individual sorts out communication based on timing, while protecting the relationship status [43]. In Indonesia, HIV related stigma and discrimination within families of the infected participants resulted into guilt consciousness among the infected individuals [44]. Also some participants experienced stigma and discrimination while accessing health care services and this influenced their decision to disclose the HIV status to other health care professionals [45]. Similarly, another study in Indonesia revealed that HIV infected people deterred to disclose their HIV status because of fear of stigma and discrimination [46].

In the present study one participant did not see the reason to disclose his HIV status to the sexual partner due to fear of hurting himself implying that avoidance of possible discomfort is important. Similarly, a study in Tanzania revealed that some participants felt less important to disclose their HIV status [47] implying that people may have personal reservations when it comes to health status disclosure. However, a multicenter study in Kenya, Namibia and Tanzania found that HIV status disclosure to a sexual partner was high, and men were more likely to disclose their status to partners [48]. Similarly Lugalla [49] reported that men were more readily to share their HIV status with spouses. On the contrary, women’s fear of abandonment and loss of economic support if they disclose their HIV status to the sexual partner is a big concern [50]. Similarly, most women feared of divorce and loss of financial support [51]. The fact that in the present study one woman was separated because of disclosing her status to the sexual partner implies the consequences may be detrimental. Similarly, Plotkin [52] revealed that in Tanzania, disclosure of HIV status ended the sexual relationship to some women. Interestingly, another study in Tanzania revealed that women who disclosed their HIV status to the sexual partners were accepted without negative outcomes [53] suggesting that HIV disclosure may have negative or positive implications.

The reported self-acceptance among the participants in the present study implies that the acquired knowledge during the trial was essential to facilitate peaceful life. Participation in daily duties suggests that regardless of HIV positive results participants were able to cope with their health status. Self-acceptance has been a great opportunity for people with HIV to cope with their illness. In Kenya, participation in HIV vaccine trial meant gaining immense knowledge and skills towards coping against challenges of stigma and discrimination [54]. In US, minority population living with HIV experienced peace with themselves and this was attributed directly to the changes in their lifestyles of which normal daily life was embraced mentally and psychologically [55]. In the present study, some participants chose not to disclose their HIV status to maintain privacy suggesting that protecting one’s status was essential to live peaceful life. Non-disclosure may allow people to normalize their lives without fear of rejection. In South Africa, self-acceptance among older adults living with HIV was important because they did not want to feel cheated out of life by the HIV diagnosis [56]. In addition, connection to the clinical trial site ensures recognition of the volunteers. In the present study, connections to the researchers post trial follow-ups appears a useful resource for accessing free medical consultations.

### Limitations and strength

Although the sample comprises of only four participants, adhering to methodological standards for qualitative research strengthened the inferences from our study. Our perspectives regarding the previous experiences of the trial volunteers may have influenced the findings but we strived to study the present participants as a unique group with prolonged experiences after acquiring HIV infection. Repeated interviews with the same participants might bring monotonous experiences but we prolonged time interval for at least two years without telling what kinds of questions will be asked next to avoid pre-perceived responses. Rigor was maintained throughout the study. The use of national language, Kiswahili during data collection allowed the participants to speak freely and made the researcher to stay close to participants own native language in identifying the codes. The triangulation of data sources (four participants each interviewed three times at different time points) and use of two researchers to code the transcripts increased trustworthiness of the findings which is important in qualitative research [57]. The direct quotes from participants’ description of their experiences are presented to allow the readers ascertain the dependability of the study findings.

## Conclusions

The article presents results from HIV infected volunteers who participated in Phase I/II HIV vaccine trials from urban Tanzania. The uncertainties experienced by the participants post trial are connected to the inadequate knowledge about HIV vaccine trials in the surrounding communities. However, informed decision, and personal commitment in the trial appear important coping resources for the participants post participation in the HIV vaccine trials. Thus, balancing controversies and coping resources facilitate normal life for the individuals who participate in HIV vaccine trials albeit infected with HIV. Information and education on HIV vaccine trials in the community surrounding trial participants is important to reduce negative comments to any volunteer who participate in HIV vaccine trials. The researchers should continue to apply principles of good participatory practice to increase awareness about the clinical trials in the settings where the trials are being conducted. Longitudinal studies among clinical trial volunteers post exit is crucial to gain more insights about the effects of uncertainties to their lives, and coping resources.

### Implications for research, practice, and training

#### Research

Longitudinal studies among clinical trial volunteers post exit is crucial to gain more insights about the effects of uncertainties to their lives, and coping resources.

#### Practice

The HIV vaccine trials’ implementers need to understand the challenges the infected volunteers may confront while coping with their health status.

#### Training

The researchers should continue to use guidelines for good participatory practice to increase awareness about the clinical trials in the settings where the trials are being conducted.

## Supporting information

S1 Checklist (PDF)
